# Commentary: Fish and the thyroid: A Janus Bifrons relationship caused by pollutants and the omega-3 polyunsaturated fatty acids

**DOI:** 10.3389/fendo.2023.1138245

**Published:** 2023-04-11

**Authors:** Silvana Hrelia, Maria Cristina Barbalace, Salvatore Cannavò, Rosaria Maddalena Ruggeri

**Affiliations:** ^1^ Department for Life Quality Studies, Alma Mater Studiorum, University of Bologna, Rimini, Italy; ^2^ Department of Human Pathology and Childhood “G. Barresi” (DETEV), University of Messina, Messina, Italy

**Keywords:** fish, Mediterranean diet, autoimmunity, endocrine disruptors, PUFA - polyunsaturated fatty acids, nutraceuticals

## Introduction

We read with interest the paper published in the journal by Benvenga S, et al. (1) about the complex interplay between thyroid disorders and the intake of omega-3 polyunsaturated fatty acids (PUFAs), whose main dietary source is represented by fish. In their review, Benvenga et al. (1) put a specific warning on the potential heavy metal content in fish and the risks deriving from its consumption, suggesting that there would be the place for the use of nutraceuticals containing omega-3 PUFAs to prevent or contrast autoimmune thyroiditis, overcoming the risk of contaminants in fish. Although pharmacological supplementation would be sometimes useful, also limiting dietary consumption to fishes containing low levels of pollutants and including fish within the framework of a healthy diet represent a good choice.

## Fish: Friend or foe?

Benvenga et al. elaborate on the adverse health effects of consuming fish containing high heavy metal levels, although they also state that most consumed fish is generally safe. Indeed, heavy metals, particularly mercury (Hg), are mostly concentrated in large predatory fishes, but their content in small fishes, like anchovy and sardine, is generally low (1). These small oily fishes (the so-called “pesce azzurro”) represent the most frequently consumed fishes in the Mediterranean diet, and their consumption is associated with the lowest risk of contamination and the higher intake of PUFAs, as the authors themselves emphasized (1). Moreover, the European Union establishes that the maximum Hg quantity in fish sold in Europe must be equal to 0.5 mg/kg of edible product (2). Salmon, anchovies, sardines, and trout contain Hg levels far from this limit. Hence, the risk of adverse effects of Hg is substantially low, strictly depending on the type of fish (3). Thus, a moderate consumption of oily fishes should be considered safe and healthy; accordingly, fish consumption is recommended twice a week in order to provide 250 mg/day of omega-3 acids [EPA + Docosahexaenoic acid (DHA)] (4). The fragmented information that consumers receive about the nutritional value and health risks associated with fish can result in confusion or misperceptions about these food sources. However, as clearly stated by Mozaffarian and Rimm (5), the benefits of fish intake largely exceed the potential risks, with the exception of a few selected species in sensitive populations.

## Fish vs. polyunsaturated fatty acid nutritional supplements

There is an argument to be made that fish has been considered a better choice than supplementation in the form of nutraceuticals in the treatment of chronic degenerative diseases. Dietary fish intake is associated with a reduction in fatal cardiovascular disease (CVD) events in a threshold manner, with a benefit seen with approximately two servings per week of fish (6), whereas multiple randomized studies and meta-analysis showed inconsistent effects on the reduction of CVD risk with low- or high-dose PUFA supplementation in people at high risk of CVD, with many studies demonstrating no evidence of benefit (7–9). In an interesting review, Petsini et al. collected and compared the existing fish intervention trials in healthy volunteers with the aim to examine the effect of either fish intake or the consumption of omega-3 fatty acids *via* capsules on biomarkers related to CVD. They concluded that not only do fish oil supplements have a beneficial effect on several CVD biomarkers but also fish consumption gives promising results, affecting CVD and inflammatory and thrombotic biomarkers, despite differences between studies and challenges in conducting/comparing dietary and supplement intervention trials (10). Indeed, the ingredients of fish flesh other than PUFAs may also contribute to the beneficial effect of its consumption, such as amino acids, trace elements, and other micronutrients, suggesting that the food in its complexity has a protective role (11–15). This point has also been stated by Jacobs et al., proposing that ‘‘thinking food first’’ results in more effective nutrition research and policy (16). Although Benvenga et al. (1) did not neglect the difference between fish consumption and omega-3 PUFA supplementation, we would like to further emphasize the importance of the whole nutritional composition of fish, which could not be simply reduced to PUFA content. However, not everyone is a seafood fan, and nutraceuticals could be a good choice in people who do not like fish, have a fish allergy, or are vegetarian, for instance.

## Fish consumption in the framework of Mediterranean diet

Undoubtedly, fish consumption should be more studied in regard to its effect on chronic inflammatory and immune-mediated disorders, including autoimmune thyroiditis, and the balance between “costs” (i.e., contaminants) and benefits (healthy components) should be carefully evaluated. Moreover, fish influence should be included in a complete healthy dietary plan, including fish with low Hg content, as patients would be benefited both from fish intake and other micronutrients and nutraceuticals. In this light, the Mediterranean diet may represent a healthy nutritional model. Indeed, fish (namely, the blue one rich in omega-3) is a cornerstone of the Mediterranean diet (17). This dietary regimen is characterized by a high intake of vegetables, legumes, fresh fruits, nuts, whole grains, and olive oil; frequent and moderate consumption of red wine; moderate intake of seafood (two times a week, preferably choosing small oily fishes), dairy products, poultry, and eggs; and a low consumption of red meat and processed meat products. Due to the synergistic effects of its many ingredients, the Mediterranean diet exerts anti-inflammatory, immunomodulatory, and antioxidant effects, which are also beneficial to the health status in the setting of autoimmune thyroiditis (18). Thus, in our opinion, fish consumption must be considered in the frame of the Mediterranean diet, and the importance of a healthy diet should not be overlooked.

## Conclusion

In conclusion, supplements represent a useful way to get nutrients otherwise lacking in selected populations and specific periods of life, but they cannot replicate all of the nutrients and benefits of whole foods and are not intended to replace a healthy diet. Tailored dietary interventions should represent a useful lifestyle strategy for preventing or counteracting the autoimmune inflammation of the thyroid gland in the long run. Obviously much research is still needed, but we believe that is possible to suggest that an optimal consumption of fish as part of a Mediterranean diet plan represents the best preventive or protective strategy against many chronic or degenerative diseases, including autoimmune thyroid diseases.

## Author contributions

HS and RMR wrote the first draft of the manuscript. MCB and CS contributed to manuscript revision. All authors read and approved the submitted version.
